# Basophil-derived IL-6 regulates T_H_17 cell differentiation and CD4 T cell immunity

**DOI:** 10.1038/srep41744

**Published:** 2017-01-30

**Authors:** Chae Min Yuk, Hyeung Ju Park, Bo-In Kwon, Sang Joon Lah, Jun Chang, Ji-Young Kim, Kyung-Mi Lee, Su-Hyung Park, Seokchan Hong, Seung-Hyo Lee

**Affiliations:** 1 Biomedical Science and Engineering Interdisciplinary Program, Korea Advanced Institute of Science and Technology (KAIST), Daejeon, 34141, Korea; 2 Graduate School of Medical Science and Engineering (GSMSE), Biomedical Research Center, KAIST Institute for the Bio-Century, KAIST, Daejeon, 34141, Korea; 3 K-herb Research Center, Korea Institute of Oriental Medicine (KIOM), Daejeon, 34054, Korea; 4 Division of Life and Pharmaceutical Science, Ewha Womans University, Seoul, 03760, Korea; 5 Department of Biochemistry and Molecular Biology, Korea University College of Medicine, Seoul, 02841, Korea; 6 Division of Rheumatology, University of Ulsan College of Medicine, Asan Medical Center, Seoul, 05505, Korea

## Abstract

Basophils are rare, circulating granulocytes proposed to be involved in T helper (T_H_) type 2 immunity, mainly through secretion of interleukin (IL)-4. In addition to IL-4, basophils produce IL-6 and tumor necrosis factor (TNF)-α in response to immunoglobulin E (IgE) crosslinking. Differentiation of T_H_17 cells requires IL-6 and transforming growth factor (TGF)-β, but whether basophils play a significant role in T_H_17 induction is unknown. Here we show a role for basophils in T_H_17 cell development by using *in vitro* T cell differentiation and *in vivo* T_H_17-mediated inflammation models. Bone marrow derived-basophils (BMBs) and splenic basophils produce significant amounts of IL-6 as well as IL-4 following stimulation with IgE crosslink or cholera toxin (CT). In addition, through IL-6 secretion, BMBs cooperate with dendritic cells to promote T_H_17 cell differentiation. In the T_H_17 lung inflammation model, basophils are recruited to the inflamed lungs following CT challenge, and T_H_17 responses are significantly reduced in the absence of basophils or IL-6. Furthermore, reconstitution with wild-type, but not IL-6-deficient, basophils restored CT-mediated lung inflammation. Lastly, basophil-deficient mice showed reduced phenotypes of T_H_17-dependent experimental autoimmune encephalomyelitis. Therefore, our results indicate that basophils are an important inducer of T_H_17 cell differentiation, which is dependent on IL-6 secretion.

Basophils, rare circulating granulocytes that account for less than 1% of peripheral blood leukocytes, are characterized by the presence of basophilic granules in the cytoplasm, and express the high affinity receptor for immunoglobulin (Ig) E (FcεRI) and CD49b (DX5)^1^,^2^,^3^. They are usually generated from granulocyte-monocyte progenitors that become basophil lineage-restricted progenitors in the bone marrow (BM)^4^. They also share many features with mast cells (MCs) including the expression of FcεRI, requirement of interleukin (IL)-3 for their development and recruitment, T helper type 2 (T_H_2) cytokine production, and the release of lipid mediators such as histamine upon activation^5^,^6^,^7^,^8^,^9^,^10^. Previously, many studies have shown that recruitment of basophils to lymph nodes (LNs) is essential and sufficient for T_H_2 cell differentiation, and basophils may function as antigen presenting cells (APCs), similar to dendritic cells (DCs) and macrophages, since they express MHC class II as well as the co-stimulatory molecules, CD80 and CD86^11^,^12^,^13^,^14^,^15^. However, these studies have been challenged by subsequent findings that both DCs and basophils are required for optimal T_H_2 responses^16^,^17^,^18^,^19^. Furthermore, depletion of basophils has little to no effects on T_H_2 immunity in experimental asthma and parasite infection models, while DC depletion results in impaired T_H_2 differentiation, which is restored by adoptive transfer of CD11c^+^ DCs^17^,^18^,^19^,^20^,^21^. Thus, the nature of basophil function in mediating T_H_ cell differentiation, and T_H_17 development in particular, remains unclear.

While T_H_17 cells protect the host against extracellular pathogens such as extracellular bacteria and fungi, these cells have also shown to contribute to the development of organ-specific autoimmune diseases^22^,^23^. The combination of IL-6 and transforming growth factor (TGF)-β has shown that the pro-inflammatory cytokine IL-6 is a potent differentiation factor for T_H_17 cells by modulating TGF-β-driven induction of Foxp3^+^ regulatory T (Treg) cells^24^. Although T_H_17 cell differentiation requires IL-6 as well as TGF-β, under many inflammatory conditions, the source of IL-6 remains unclear. Therefore, we focused on identifying the source of IL-6 during the differentiation of naïve CD4 T cells into the T_H_17 cells.

A recent study suggests that MCs and basophils play a role in antigen-induced arthritis^25^. Further, human basophils have shown to interact with memory CD4 T cells in T_H_17-associated diseases including inflammatory bowel diseases (IBDs) through induction of basophil-derived histamine and histamine receptors on T cells^25^,^26^. In addition, IL-3 released by CD4 T cells activates basophils and can aggravate collagen-induced arthritis (CIA)^27^. In addition to IL-4, basophils secrete other pro-inflammatory cytokines such as IL-6 and tumor necrosis factor (TNF)-α^28^, which indicates that basophils may be involved in the induction of T_H_17 cell-mediated immune responses. To evaluate whether basophils could mediate T_H_17 cell differentiation, we designed two different approaches; the first is an *in vitro* T_H_ differentiation system using naïve CD4 T cells, and the other is *in vivo* inflammation models using cholera toxin (CT), a potent mucosal adjuvant-mediated lung inflammation and experimental autoimmune encephalomyelitis (EAE), an animal model of multiple sclerosis. Here we demonstrate that basophils augment T_H_17 cell differentiation through their cytokine production, and enhance T_H_17-mediated immune responses in a CT-induced lung inflammation and EAE models.

## Results

### Characterization of and cytokine production by BM-derived basophils (BMBs)

**Figure 1:**
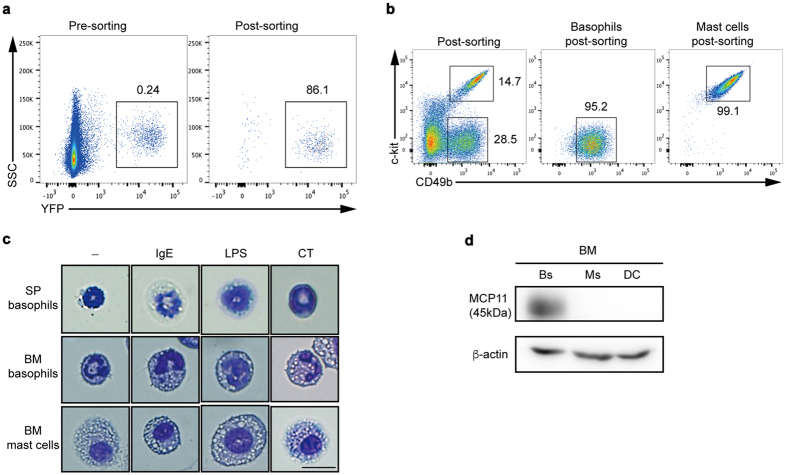
Sorting strategy and morphology of SPBs, BMBs, and BMMCs. (**a**) YFP^+^ basophils (SPBs) from 6-week-old spleens of *Mcpt8-cre-*YFP mice were sorted and the purity was analyzed by flow cytometry (n = 3). **(b)** BM-derived c-kit^-^CD49b^+^ basophils and c-kit^+^ CD49b^+^ MCs were isolated and the purity of each population was analyzed by flow cytometry. **(c)** Isolated SPBs, BMBs, and BMMCs were stimulated with IgE, LPS, or CT, and stained with Giemsa followed by analysis by light microscope (400x, scale bar = 10 μm). **(d**) 4 × 10^6^ cell lysates from BMB (Bs) for 1-week culture, BMMCs (MC) for 4 weeks culture, and BMDCs (DC) for 1-week culture were subjected to western blot analysis for MCP-11 protein expression. Full-length blots are presented in Supplementary Figure 1a. Data are representative of three independent experiments (n = 3).

In the search for mediators of T_H_17 cell induction, we focused on basophils since they are recruited to the site of inflammation and once activated, secreting large amounts of IL-6. To assess the exact roles of basophils in T_H_17 cell differentiation *in vitro*, we first isolated YFP^+^ splenic basophils (SPBs) from *Mcpt8-cre-*yellow fluorescence protein (YFP) mice^29^ (Fig. 1a). We also differentiated BM stem cells into basophils (BMBs) using the IL-3 growth factor over 7 to 9 days in culture^7^ and mast cell (BMMCs) cultured with IL-3 at least for 4 weeks. Because YFP is not expressed in BMBs as it is in SPBs (data not shown), BMBs cells were identified by their lack of c-kit expression and by expression of CD49b (c-kit^−^CD49b(DX5)^+^) (Fig. 1b). SPBs also express CD49b (DX5) (data not shown). BMMCs can be distinguished from BMBs by the expression of c-kit (Fig. 1b). We further compared the morphology of purified YFP^+^ cells with BMBs and BMMCs to determine whether YFP^+^ cells were true basophils. Following stimulation of purified cells with IgE, LPS, or CT, analysis of cell morphology showed that both populations of basophils contained basophilic granules and typical bilobed nuclei while MCs had many granules and uniformed nucleus, and were bigger than basophils (Fig. 1c). Since basophils are known to highly express the tryptase like mast cell protease 11 (MCP11) but MCs do not^30^, we examined whether isolated BMBs, BMMCs, and BMDCs express MCP11. To this end, BMBs express 42-kDa mMCP11, but neither BMMCs nor BMDCs do (Fig. 1d), indicating that c-kit^−^CD49b(DX5)^+^ cells are genuine basophils and differ from MCs in their phenotypes and morphology.

**Figure 2:**
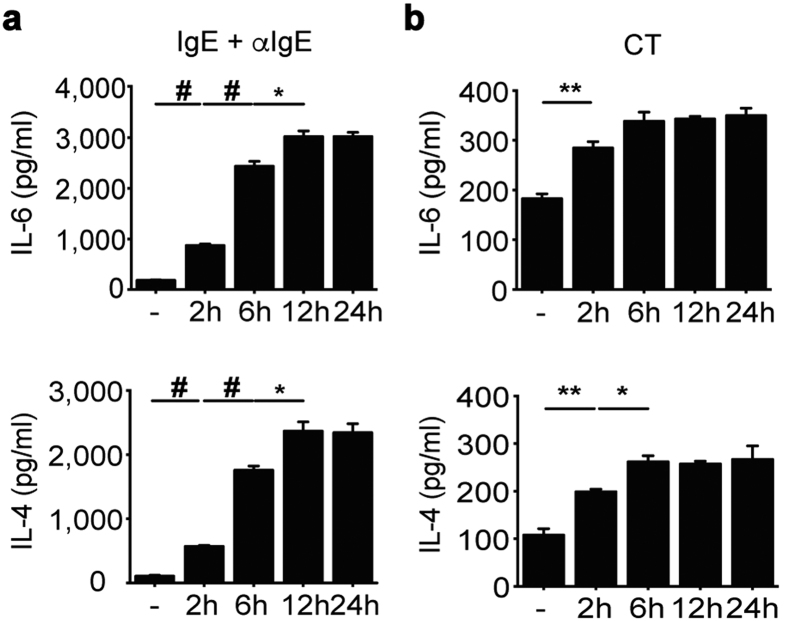
IL-6 and IL-4 production by BMBs with various stimulations. FACS-sorted BMBs were stimulated with IgE crosslinking **(a)** or CT **(b)** in a time-dependent manner. Secretion of IL-4 and IL-6 was quantified. Data are representative of three independent experiments (n = 3).

Prior studies have shown that once activated by IgE crosslinking, proteases, or cytokines (e.g. IL-3 and IL-33), basophils can release IL-4 and IL-6 in large quantities^28^. In agreement with these findings, we found that after stimulation with IgE and anti-IgE antibody, BMBs secreted large amounts of IL-6 as well as IL-4 in a time-dependent manner (Fig. 2a). Similar results were obtained when BMBs were treated with CT (Fig. 2b). Likewise, SPBs produced substantial amounts of IL-6 and IL-4 following stimulation with CT in the presence of IL-3 (Supplemental Fig. S1b). Thus, these results indicate basophils mediate substantial enhancement of IL-6 and IL-4 production in response to CT as well as IgE crosslinking.

### Induction of T_H_17 cell differentiation by BMBs through IL-6 secretion

**Figure 3:**
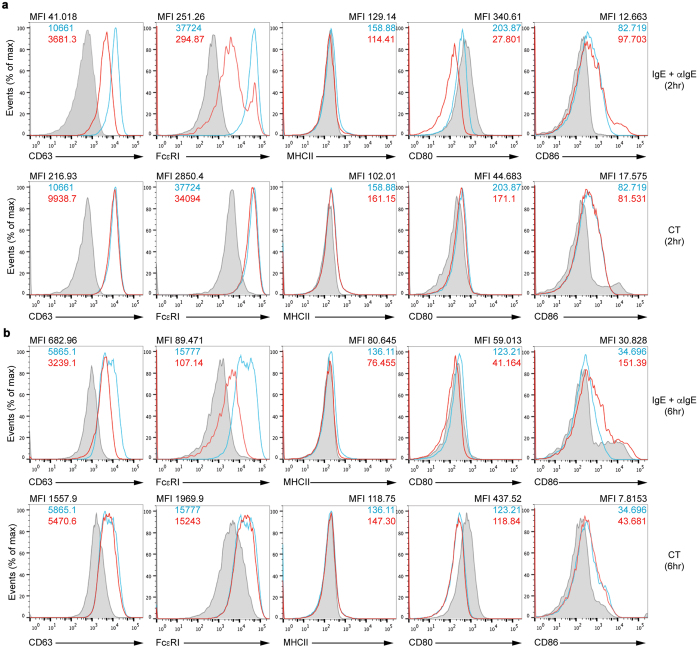
Expression of cell surface molecules on BMBs. Flow cytometric analysis of CD63, FcεRI, MHCII, CD80, and CD86 expression on BMBs was performed after IgE crosslinking (**a** and **b**, upper red line) or CT stimulation (**a** and **b**, lower red line) for 2 hours **(a**), 6 hours (**b**) or without stimulation (blue line). Grey bars represent staining with isotype control antibody. Data are representative of four independent experiments.

Next, we investigated whether basophils could induce differentiation of T_H_17 cells. Firstly, to examine the possibility that basophils function as APCs, we analyzed expression of MHC class II and co-stimulatory molecules along with other markers on BMBs and YFP^+^ SPBs activated through IgE crosslinking or CT stimulation for both 2 and 6 hours. We detected expression of CD63, a degranulation marker that is specific for basophils and MCs, which showed reduced expression by IgE crosslinking after 2 or 6 hours but not by CT stimulation (Fig. 3). MHC class II was not expressed on BMBs and expression of CD80 was even inhibited after stimulation overall. However, expression of CD86 co-stimulatory molecule was slightly increased especially on 6 hour-IgE stimulated BMBs (Fig. 3). We also confirmed that YFP^+^ SPBs expressed these molecules similar to BMBs (Supplemental Fig. S2). In YFP^+^ SPBs, the expression of co-stimulatory molecule especially CD86 was enhanced with either stimulation. Further, although MHC class II was expressed on YFP^+^ SPBs, their expression was not enhanced with stimulation. Thus, these results indicate that activated basophils may express co-stimulatory molecule such as CD86, which can induce activation of T cells.

**Figure 4:**
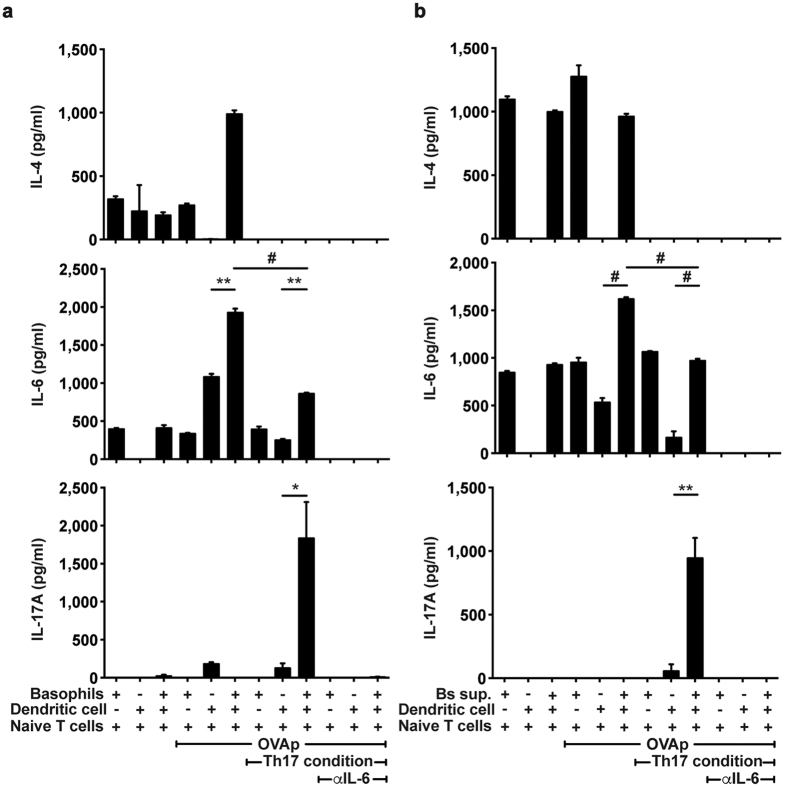
Cooperation of basophils and DCs to drive T_H_17 cell differentiation. (**a**) Production of IL-4, IL-6, and IL-17A following co-culture of IL-6 deficient naïve OT-II T cells with DCs, basophils or both in the presence of OVA peptides or with OVA peptides plus T_H_17 differentiation conditions was measured. (**b**) Supernatants of basophils stimulated by IgE crosslinking for 6 hours were added into each co-culture condition. After 3 days of culture, the levels of IL-4, IL-6, and IL-17A were quantified from supernatant. Data are representative of three independent experiments (n = 3) and are presented as mean ± SEM.

Because activated basophils express co-stimulatory molecules, and secrete IL-6, we next investigated the possibility that basophils mediate T_H_17 cell differentiation. Isolated BMB (c-kit^−^CD49b^+^) were activated either by IgE crosslinking or with cytokines and then were co-cultured with naïve IL-6 deficient OT-II T cells for 3 days with or without CD11c^+^ BMDCs in the presence or absence of OVA peptides. We found that in the presence of OVA peptides, IL-6 and IL-4 concentrations were increased in co-cultures of basophils, DCs and naïve CD4 T cells when compared to either basophils with T cells or DCs with T cells (Fig. 4a). Naïve CD4 T cells were stimulated with OVA peptides under T_H_17 differentiation conditions in which TGF-β, and anti-IL-4 and anti-IFN-γ antibodies except for IL-6 were included. In the presence of basophils and DCs, concentrations of IL-17A were significantly higher than in the presence of basophils or DCs alone (Fig. 4a). Furthermore, neutralizing IL-6 using blocking antibodies inhibited IL-17A production (thus, T_H_17 cell differentiation) by T cells. Together these findings indicate that neither basophils nor DCs can induce T_H_17 cell differentiation in the absence of IL-6, but basophils can augment differentiation of naïve CD4 T cells into T_H_17 cells in the presence of DCs by providing the required cytokine.

To evaluate whether secreted factors derived from basophils are responsible for T_H_17 cell differentiation, supernatants of activated basophils were used to stimulate T cells in the presence or absence of DCs. Consistent with the data above, we found that IL-17A was highly secreted in the co-culture of DCs and naïve T cells with the supernatant in response to OVA peptides (Fig. 4b) indicating that basophil-derived factors induced T_H_17 cell differentiation. Furthermore, addition of anti-IL-6 antibody suppressed secretion of IL-17A, or T_H_17 cell differentiation (Fig. 4b). Thus, these findings indicate IL-6 produced by basophils represents a key cytokine that is sufficient to mediate T_H_17 cell differentiation. Similarly, we found that naïve T cells proliferated in response to OVA peptides and T_H_17 conditions when compared to the other cell co-cultures (Supplemental Fig. S3a). It has been previously reported that basophils could not induce T cell proliferation because IL-4 and IL-6 secreted from basophils show an inhibitory effect on proliferation of CD4 T cells^16^,^31^. However, our results show that supernatants from activated basophils could enhance proliferation of CD4 T cells though basophil itself not (Supplemental Fig. S3). Thus, these results suggest basophils may disturb CD4 T cell proliferation even though they induce differentiation of T_H_17 cell from naïve CD4 T cells.

### Cholera toxin-mediated T_H_17 inflammation in IL-6 deficient mice

**Figure 5:**
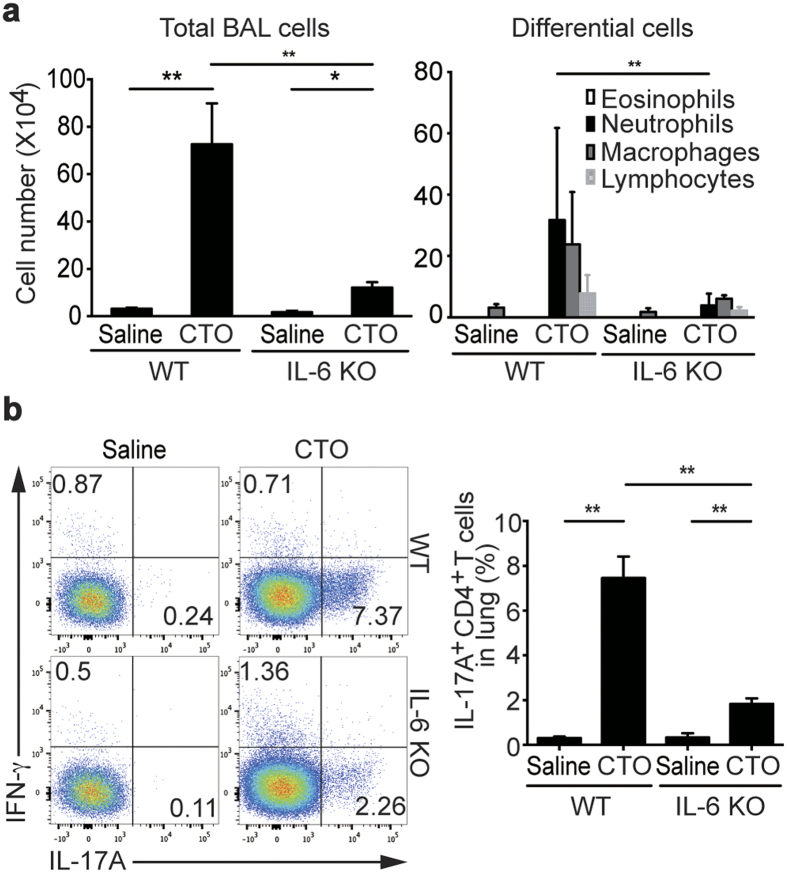
Reduced CT-mediated T_H_17 responses and IL-17A production in absence of IL-6. (**a**) After immunization with intranasal CTO of 6 to 8- week-old WT or IL-6 deficient mice on the B6 background, total cell and inflammatory cell numbers were assessed in BAL fluid. (**b**) Representative and compiling flow cytometric analysis of IL-17A and IFN-γ production by CD4 T cells from lungs of WT and IL-6 deficient mice are depicted. Data are representative of four independent experiments (n = 5) and are presented as mean ± SEM.

Next, we examined whether basophils and/or IL-6 secreted from basophils are important mediators of T_H_17-induced inflammation *in vivo*. CT, a major exotoxin produced by *Vibrio cholerae*, is a potent mucosal adjuvant. Recent studies have shown that intranasal administration of CT as a mucosal adjuvant induces strong, acute T_H_17 responses as bystander antigens^32^,^33^. Therefore, we utilized the CT-induced T_H_17 cell-mediated inflammatory model to demonstrate the role of basophils during the induction of T_H_17 cells. First, to confirm IL-6 is critical for the induction of T_H_17 responses, we challenged wild-type (WT) and IL-6 deficient mice with CT plus chicken egg ovalbumin (CTO). Both total cell numbers in bronchoalveolar lavage (BAL) fluid and neutrophil recruitment were enhanced in WT mice challenged with CTO, while these responses were significantly decreased in IL-6 deficient mice (Fig. 5a). We next used flow cytometric analysis to examine IL-17A and IFN-γ production by CD4 T cells isolated from the lung. Following CTO administration, the frequencies of IFN-γ producing CD4 T cells were not different between WT and IL-6 deficient mice, whereas IL-17A producing CD4 T cells were significantly reduced in IL-6 deficient mice (Fig. 5b). IL-17A production by CD4 T cells from draining LNs was slightly decreased but statistically not significant in IL-6 deficient mice than in WT mice (data not shown). Together these results suggest that IL-6 is critical for the induction of T_H_17 responses by CTO.

### Requirement of basophils for T_H_17 immunity

**Figure 6:**
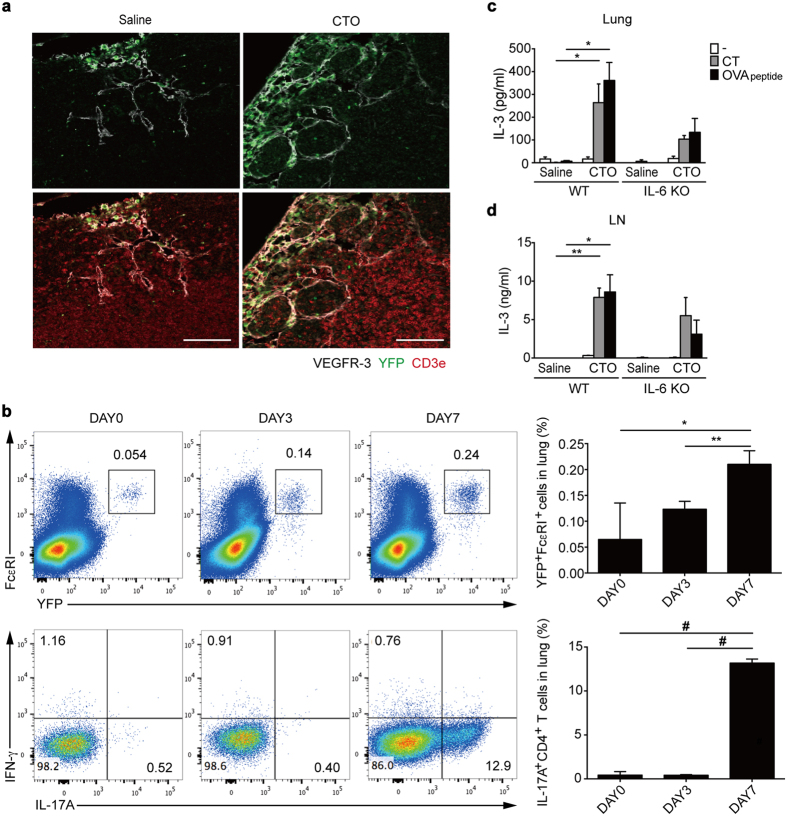
Increased basophil recruitment into draining LNs and lungs following CTO challenge. (**a**) Representative immunofluorescence confocal microscopic analysis of LNs from saline- (left) or CTO (right)-immunized 6 to 8-week-old *Mcpt8-cre*-YFP mice. Basophils (YFP-green), lymphatic vessels (VEGFR-3-white), and T cells (CD3e-red) were visualized (scale bar = 100 μm). (**b**) Representative and compiling flow cytometric analysis of basophil recruitment (FcεRI^+^ YFP^+^) in lungs at 0, 3, and 7 days after intranasal CTO challenge. **(c**,**d**) IL-3 production by CT or OVA peptide re-stimulated total lung **(c)** and LN (**d**) cells after intranasal CTO challenge (n = 3). Data are representative of three independent experiments (n = 3) and are presented as mean ± SEM.

To understand the role of basophils in the induction of T_H_17 responses, we next examined the recruitment of basophils to draining LNs and lungs in CTO-challenged mice. To this end, we quantified YFP^+^ basophils in LNs and lungs from CTO-challenged *Mcpt8-cre*-YFP mice. We collected LNs for staining of CD3^+^ T cells and lymphatic vessels at day 3 after CTO administration since recruitment of basophils to the draining LNs is known to peak at this time point^14^,^28^. Increased YFP^+^ basophil recruitment was observed within subcapsular sinus of lymphatic vessels in CTO challenged group compared to the saline group (Fig. 6a). In addition, recruitment of YFP^+^ FcεRI^+^ basophils into inflamed lungs was augmented in a time-dependent manner, peaking at day 7 following CTO administration, which was accompanied by a significant induction of T_H_17 responses in the lungs (Fig. 6b). Thus, these results show that basophils may play a role in T_H_17 responses in draining LNs and target organs.

As mentioned earlier, IL-3 released by CD4 T cells activates basophils. Thus, to determine a molecular factor for the recruitment of basophils^27^, we analyzed lung cells to examine the production of IL-3. For this purpose, total lung and LN cells either were left unstimulated or were re-stimulated with OVA peptides. Significantly higher amounts of IL-3 were detected from total LNs and lungs following CTO-challenged WT mice than IL-6 deficient mice (Fig. 6c,d). Thus, these results suggest that IL-3 secreted from lung cells induces recruitment of basophils into the lungs.

### Reduced induction of CT-mediated T_H_17 responses in basophil-deficient mice

**Figure 7:**
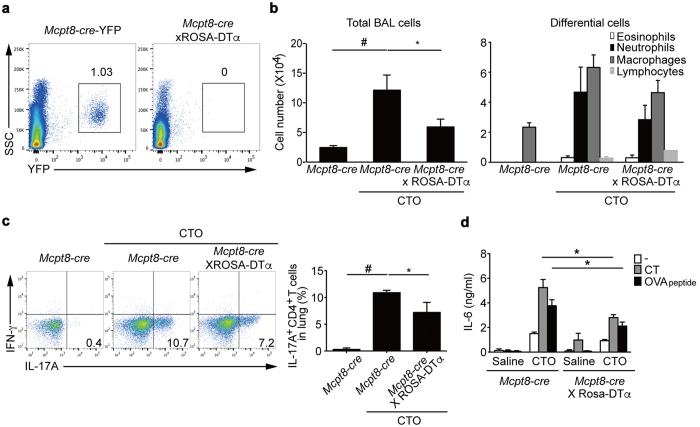
Reduced T_H_17 responses in basophil-deficient mice. **(a**) Representative flow cytometric analysis of the presence of basophils in spleens of 6 to 8-week-old *Mcpt8-cre-*YFP and basophil-deficient mice (gating on YFP^+^ cells). (**b**) After immunization, total and differential inflammatory cell numbers were quantified in BAL fluid (n = 11). **(c)** Representative and compiling flow cytometric analysis of intracellular cytokine staining of CD4 T cells from lungs of CTO-immunized *Mcpt8-cre* and basophil-deficient mice indicate levels of IL-17A production. **(d**) IL-6 production by CT or OVA peptide re-stimulated total lung cells after intranasal saline or CTO challenge (n = 3). Data and are presented as mean ± SEM.

To investigate the effects of basophils on T_H_17 cell differentiation *in vivo*, we next generated basophil-deficient mice by crossing *Mcpt8-cre-*YFP mice^29^ with ROSA-DTα mice^34^,^35^. The *Mcpt8-cre*-YFP × ROSA-DTα mice showed highly efficient depletion of basophils, as shown by lack of YFP^+^ cells (Fig. 7a). To determine whether CTO-induced T_H_17 responses occur in basophil-deficient mice, we administrated CTO intranasally into *Mcpt8-cre*-YFP and *Mcpt8-cre*-YFP × ROSA-DTα mice. At the day 7 after immunization, there was a significant decrease in the total BAL fluid cells of basophil-deficient mice following CTO challenge compared to basophil-sufficient mice (Fig. 7b). Neutrophil numbers in BAL fluid of basophil-deficient mice also showed a lower trend compared to that of basophil-sufficient mice. CD4 T cells from the lungs of basophil-deficient mice exhibited significantly lower IL-17A production compared to that of basophil-sufficient mice (Fig. 7c). Finally, to assess IL-6 secretion after CTO immunization, we collected culture supernatants of total lung cells re-stimulated with either CT or OVA peptides and quantified the levels of IL-6. Production of IL-6 was significantly diminished in the absence of basophils compared to basophil-sufficient lung cells after re-stimulation (Fig. 7d). Thus, CTO-mediated T_H_17 responses are reduced in basophil-deficient mice consistent with their diminished production of IL-6.

We next investigated the impact of CD11c^+^ DCs on T_H_17 induction in response to CTO because a role for DCs in T_H_17 responses has been reported before^36^. Because treatment of transgenic CD11c-DTR mice with diphtheria toxin (DT) is lethal, BM chimeras were generated using CD11c-DTR BM to reconstitute CD45.1 WT mice^37^. There was no difference in total cell numbers or infiltrated neutrophils, the dominant inflammatory cells, in the BAL fluid of CD11c-DTR chimeric mice compared with WT chimeric mice at 7 day after CTO administration (Supplemental Fig. S4). Furthermore, IL-17A production by CD4 T cells in the lungs and draining LNs did not differ between two groups. These data indicate that classical CD11c^+^ DCs might not be critical for the induction of T_H_17 responses mediated by CTO. Collectively, these data indicate that basophils, but not DCs, play an important role in both T_H_17 differentiation and T_H_17 responses.

### Reduced EAE incidence and severity in basophil-deficient mice

**Figure 8:**
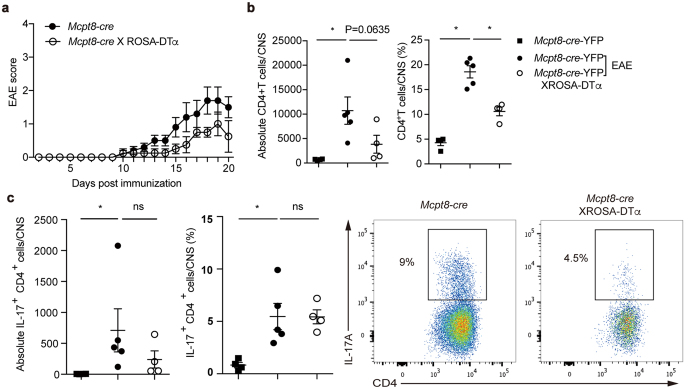
Basophil deficiency reduced EAE incidence and severity. **(a**) Clinical scores of *Mcpt8-cre-*YFP and basophil-deficient mice after EAE induction. **(b**) Frequency and absolute number of CD4 T cells in the CNS of *Mcpt8-cre-*YFP and basophil-deficient mice at 21 days post immunization. **(c**) Frequency and absolute number of IL-17A producing CD4 T cells from EAE induced mice. Data are representative of three independent experiments are presented as mean ± SEM (n = 5 for *Mcpt8-cre-*YFP mice, n = 4 for basophil-deficient mice).

To demonstrate whether basophils affect a physiologically relevant consequence, we assessed T_H_17-dependent EAE development, a mouse model of human multiple sclerosis in basophil-deficient mice. Basophil-deficient mice showed reduced EAE severity (Fig. 8a). In the central nervous system (CNS) including spinal cords and brain, basophil-deficient mice showed significantly lower infiltrated CD4 T cells compared to basophil-sufficient mice (Fig. 8b). The reduction of absolute numbers of CD4 T cells in CNS without basophils indicate a defect in T_H_17 responses resulting in diminished development of EAE. Indeed, the fewer CD4 T cells that were able to infiltrate into the CNS were functionally defective in that reduced production of IL-17A (Fig. 8c). Together, these results indicate that basophils are important for T_H_17 cell differentiation, allowing T_H_17 cells to migrate to the site of inflammation mediating pathogenic functions.

### Influence of basophil-derived IL-6 on T_H_17-mediated inflammation

**Figure 9:**
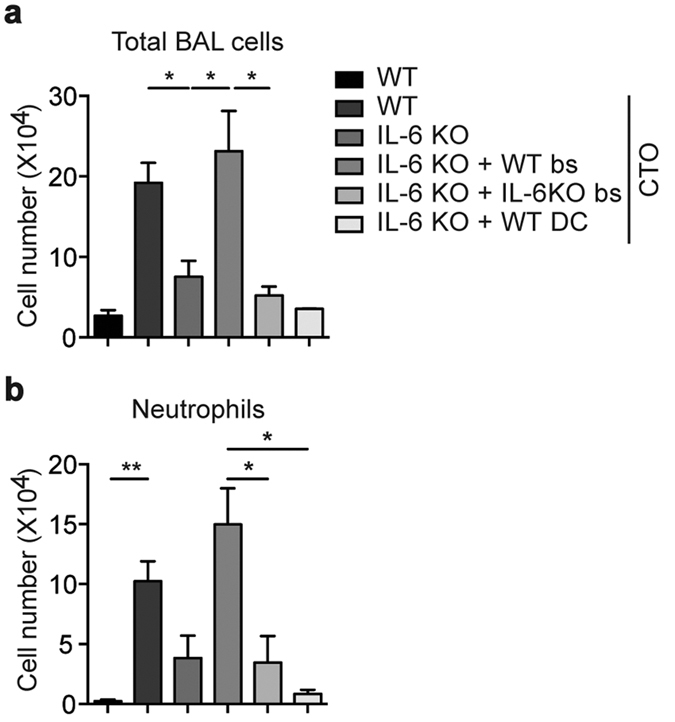
Reconstitution of basophils in CTO-mediated lung inflammation. Quantification of total inflammatory cells (**a**) and neutrophils (**b**) in BAL fluid from WT or IL-6 null mice that received WT or IL-6 deficient basophils or WT DCs after CTO administration. Data are representative of three independent experiments (n = 3) and are presented as mean ± SEM.

The above data indicate that recruited basophils are critical for the induction of T_H_17 responses. Finally, to determine whether IL-6 secreted from basophils is critical for T_H_17 responses, we performed adoptive transfer experiments in which WT BMBs, IL-6-deficient BMBs, or WT DCs were transferred into IL-6 null mice. Mice reconstituted with WT basophils exhibited CTO-mediated lung inflammation in a manner similar to that of CTO-challenged WT mice (Fig. 9a). However, in a manner similar to IL-6 deficient mice, reconstitution with IL-6 deficient basophils or WT CD11c^+^ DCs resulted in significantly less T_H_17-mediated inflammatory responses compared to WT and WT basophil-reconstituted IL-6 deficient mice (Fig. 9a). Lastly, neutrophil recruitment into the BAL fluid of IL-6 deficient basophil-reconstituted or WT CD11c^+^ DC-reconstituted IL-6 null mice was also diminished compared to WT mice and IL-6 deficient mice reconstituted with WT basophils (Fig. 9b). Altogether, our study demonstrates that basophils play a critical role in T_H_17 cell differentiation through the secretion of IL-6.

## Discussion

In this study, we investigated the role of basophils in T_H_17 cell differentiation and type 17 immunity using an *in vitro* T cell culture system and *in vivo* inflammation models. Using the *in vitro* culture system, we found that IL-17A production by naïve CD4 T cells was dramatically enhanced when co-cultured with a combination of basophils and DCs under T_H_17 differentiation conditions. Further, this finding extended to the co-culture of naïve CD4 T cells with supernatants derived from activated basophils. In the CTO-mediated lung inflammation model it was confirmed that T_H_17 responses and/or neutrophil-dominant lung inflammation peaks as basophils are recruited into lung after intranasal delivery of CTO. In addition, T_H_17 responses were found to be markedly reduced under either basophil or IL-6 deficient conditions. Lastly, the severity and incidence of T_H_17-dependent EAE were significantly diminished in the absence of basophils. Therefore, we demonstrate that basophils play an important role in T_H_17 cell differentiation primarily by secreting IL-6 in a cell-contact independent manner.

IL-6 and TGF-β are well-known T_H_17 cell differentiation factors^38^. It has been shown that not only DCs but also other cells including macrophages, fibroblasts, endothelial cells and T cells produce IL-6^39^. Although a role for DCs in the induction of T_H_17 responses has been reported^40^, the exact source of IL-6 has remained unknown. In earlier studies, it was reported that IL-4 secreted by basophils is essential for the T_H_2 cell differentiation; however, basophils can secrete not only IL-4 and TNF-α, but also IL-6 in response to papain or helminthes^12^,^14^,^28^. Furthermore, the exact contribution of basophils to T_H_2 differentiation and type 2 immune responses has been controversial^16^,^17^,^18^. Recently it was demonstrated that basophils can function as effector cells for type 2 immunity, but are not essential for T_H_2 cell differentiation. On the other hand, it has been reported that human and murine basophils are recruited into sites of T_H_17-mediated inflammation in IBD and CIA through IL-33 or IL-3 secretion, respectively^26^,^27^. Other studies also showed that basophils are recruited into joint regions in patients with rheumatoid arthritis (RA), especially juvenile RA^41^, and the disease severity is ameliorated in IL-3 deficient mice with CIA through reduced development and recruitment of basophils^27^. Therefore, we focused on IL-6 production by basophils in response to IgE crosslinking or CT stimulation since we postulated that basophils might be implicated in T_H_17 responses through IL-6 secretion.

Because basophils are rare and express surface markers common to MCs (FcεRI and CD49b (DX5)) they are not easily identified or isolated. For this reason, we utilized basophil reporter mice in which the basophil-specific marker, *mcpt8* is co-expressed with YFP along with *cre* recombinase^29^. In these mice, it is possible to isolate YFP^+^ basophils from the spleen; however, it should be noted that staining with anti-c-kit and CD49b antibodies followed by sorting is required to isolate BMBs since they do not express YFP *in vitro* development. This indicates that SPBs and BMBs might be functionally different and this remains to elucidate. Nevertheless, examination of SPBs and BMBs revealed bilobed nuclei and basophilic granules, and the production of the trypsin-like protease, MCP-11, as described previously^30^. After stimulation with IgE crosslinking or CT, these isolated basophils secreted significant amounts of IL-6 than IL-4. We postulate that the slight increase in IL-4 and IL-6 seen in the BMBs without stimulation condition is due to that they are already activated by IL-3 during the *in vitro* differentiation culture. Thus, basophils appear to respond to CT directly and subsequently secrete IL-6 and IL-4; however, the expression of a CT receptor on basophils and the CT-mediated signaling pathways remain to investigate. Furthermore, *in vitro* analysis of sorted basophils revealed basophils do not enhance expression of MHC II but CD86 co-stimulatory molecule, although it remains unclear whether these cells can function as APCs.

Our study shows that factors secreted by basophils are responsible for the induction of T_H_17 responses. Culture of naïve CD4 T cells with supernatants from activated basophils *in vitro* induced differentiation of T_H_17 cells in a cell contact-independent manner (Fig. 4b). Thus, we propose that basophils function mainly as providers of cytokines while DCs act as APCs. Moreover, proper antigen-specific T_H_17 induction requires cooperation of basophils and DCs as shown by our finding that while basophils express MHC class II and co-stimulatory molecules, they cannot mediate T cell proliferation (Supplemental Fig. S3) whereas DCs promote CD4 T cell proliferation^16^,^31^. In our *in vitro* study, we observed that IL-6 production was highly induced following co-culture of basophils with naïve CD4 T cells stimulated with OVA peptides compared to culture of basophils alone. Therefore, we postulate that basophils might be further activated by IL-3 secreted by antigen-stimulated CD4 T cells to build up a positive feedback mechanism for activation of basophils and T cells. Consistent with a previous study showing the restriction of T_H_1 responses in experimental murine colitis due to basophil-derived IL-4 and IL-6^42^, we could not detect IFN-γ production *in vitro* co-culture (data not shown). These *in vitro* data, especially that from the co-culture experiments with IL-6 deficient T cells and DCs suggest that DCs alone are unable to mediate differentiation of T_H_17 cells. Rather, basophil-derived IL-6 is necessary for optimal T_H_17 development.

For our *in vivo* functional studies, we used a CTO-induced lung inflammation model to address the role of basophils in T_H_17 immunity. Recent studies have shown that CT elicits T_H_2 responses by inducing T_H_2-associated cytokines including IL-4, IL-5 and IL-10 in mucosal tissue^43^,^44^,^45^. However, studies in several murine models have reported that CT can promote differentiation of T_H_17 cells and induce strong T_H_17 responses by acting as a bystander antigen, especially when administered intranasally^32^,^33^. Moreover, these studies have also shown that IL-17 produced by T_H_17 cells contributes to the mucosal adjuvant activity of CT. Therefore, we utilized a CTO-induced T_H_17 cell-mediated inflammatory model to demonstrate the role of basophils during the induction of T_H_17 cells. Of note, because the basophils we focused on have a short life span, we needed to demonstrate the role of these cells in an acute inflammation model. To deplete basophils from mice, we crossed basophil reporter mice with ROSA-DTα mice, which induces basophil-specific expression of DT. Consistent with *in vitro* data, we found that CTO-induced T_H_17 responses were reduced in the absence of basophils. Previously, it was reported that IL-3 derived from CD4 T cells could activate and recruit basophils into LNs following parasite infection^10^,^46^. As shown here, basophils transiently enter lymphatic vessels surrounding the cortex, but did not enter the T cell zones of the draining LNs (Fig. 6a). In addition, we observed the recruitment of basophils along with CD4 T cells producing IL-17A into target tissues 7 days after immunization. These results imply that infection can induce the generation of basophils and these newly generated basophils migrate to target organs in a manner dependent on IL-3 and/or other factors (including chemokines) from CD4 T cells^10^.

We also confirmed that basophils have a role in T_H_17-mediated autoimmune diseases such as EAE. Basophil-deficient mice show a reduced phenotype of the autoimmune disease through diminished incidence and lower infiltration of CD4 T cells. In this study, the impact of basophil deficiency in autoimmune disease model indicates the physiological relevance of the function of basophils in the infiltration to the target sites and differentiation of T_H_17 cells. We also found that IL-6 production was significantly diminished after CTO immunization in basophil-deficient mice. Reconstitution of IL-6 deficient recipient mice with WT or IL-6 deficient basophils revealed that acute lung inflammation was restored only by WT basophils. Therefore, although a variety of cells can produce IL-6, basophils play an essential role in CTO-mediated T_H_17 inflammation, and IL-6 produced by basophils affects the early phase of T_H_17 differentiation. Expanding on these results, future studies will focus on the influence of basophils on T_H_17 responses during chronic autoimmunity and extracellular pathogen infection models.

In summary, we investigated the roles of basophils during T_H_17 cell differentiation using both *in vitro* T_H_ cell differentiation, and CTO- and autoimmune-mediated murine T_H_17 inflammatory models. We found that IL-6 produced by activated basophils is involved in the induction of differentiation of naïve CD4 T cells into T_H_17 cells as well as T_H_17 responses in both *in vitro* and *in vivo*. Further, our results demonstrate that basophils are recruited to target sites during CTO-mediated lung inflammation. Although the percentage of blood basophils is low, IL-6 produced by activated basophils may be substantial compared with other cells do. Thus, we believe that basophils are necessary to induce differentiation of T_H_17 cells. In conclusion, the present study provides evidence that basophils play a crucial role in T_H_17 immunity through the secretion of IL-6.

## Methods

### Mice

*Mcpt8*-*cre***-**YFP (C.129S4(B6)-Mcpt8^tm1(cre)Lky^/J) mice^29^ and WT C57BL/6, ROSA-DTα, C57BL/6 J background IL-6 deficient, CD11c-DTR, CD45.1 congenic and OT-II mice were purchased from Jackson Laboratory (Bar Harbor, ME). IL-6 deficient OT-II mice were obtained by crossbreeding. All animals maintained under specific pathogen-free condition. All mice were fed with a standard normal diet (PMI Lab Diet) *ad libitum* with free access to water and 6 to 10 week old male mice were used for this study. All animal protocols were approved by the Animal Care Committee of Korea Advanced Institute of Science and Technology (KAIST, KA2015-25), and all experimental procedures were performed in accordance with the relevant guidelines and regulations by KAIST.

### BMB induction

Cell cultures were performed in RPMI 1640 medium supplemented with penicillin G (50 U/ml), 50 μg/ml of streptomycin, 2 mM L-glutamine (Invitrogen, Grand Island, NY), and 10% fetal bovine serum (WelGene, Seoul, Korea) at 37 °C in a humidified atmosphere containing 5% CO_2_. IL-3 (10 ng/ml, Peprotech, Rocky Hill, NJ) containing medium was added to BM stem cell culture for 7 to 9 days.

### Sorting and activation of BMBs and SPBs

After BM culture, DX5(CD49b)^+^/c-kit^+^ MCs and DX5(CD49b)^+^/c-kit^−^ basophils were isolated using a FACSAria II (BD biosciences, San Jose, CA). YFP^+^ basophils were isolated from splenocytes of *Mcpt8*-cre-YFP mice using a FACSAria II. After sorting, 0.5 × 10^5^ BMBs and SPBs were activated with IgE (clone 27-74, 10 μg/ml, BD biosciences, San Diego, CA) and anti-IgE (clone RME-1, 10 μg/ml, BioLegend, San Diego, CA) crosslinking, LPS (100 ng/ml, In vivogen, San Diego, CA), or CT (100 ng/ml, List Biological Laboratories, Campbell, CA) for 2 hours. IL-4 and IL-6 were measured with sandwich ELISA^47^ (BD biosciences).

### Flow cytometry of activated BMBs and SPBs

Activated basophils for 2 hours were pre-incubated for 15 minutes on ice with Fc-block (clone 2.4G2) and stained with PerCP-Cy5.5-labeled anti-*c-kit* (clone 2B8), PE-Cy7-labeled anti-CD49b (clone DX5), PE-eFluor610-labeled anti-FcεRI (clone MAR-1), PE-labeled anti-CD63 (clone NVG-2), APC-labeled anti-I-A/I-E (M5/114.15.2), APC-Cy7-labeled anti-CD86 (clone GL-1), and BV650-labeled anti-CD80 (clone 16-10A1) for assessment of surface molecule expression. All antibodies were purchased from BD biosciences.

### Isolation and co-culture of lymphocytes with basophils and DCs

Naïve CD4 T cells stained with APC-labeled anti-CD4 (clone RM4-5) and FITC-labeled anti-CD62L (clone MEL-14) were isolated, with purity of over 98%, from IL-6 deficient OT-II mice using a FACSAria II. CD49b(DX5)^+^ c-kit^−^ basophils were isolated, with purity higher than 95%, from the BM culture supplemented with IL-3 (10 ng/ml, Peprotech) for 7–9 days using a FACSAria II. Conventional CD11c^+^ DCs were isolated, with purity higher than 90%, from BM cells cultured with GM-CSF (20 ng/ml, Peprotech) using CD11c microbeads (Miltenyi Biotec, Bergisch-Gladbach, Germany). 2.5 × 10^4^ CD4 T cells were cultured with 0.5 × 10^4^ basophils activated with IgE (10 μg/ml, BD biosciences) and anti-IgE (10 μg/ml, BioLegend) and mature CD11c^+^ DCs for 3 days. 10 μM of OVA_323–339_ peptide (ISQAVHAAHAEINEAGR), anti-IL-4 (10 μg/ml, clone 11B11, BD biosciences), anti-IFN-γ (10 μg/ml, clone XMG1.2, BD biosciences), anti-IL-6 (5 μg/ml, clone MP5-20F3, BD biosciences), and TGF-β (5 ng/ml, Peprotech) were added as indicated. Cell culture was performed in 96-well round-bottomed plates in a total volume of 200 μl.

### Immunoblotting

Cells were lysed in RIPA buffer (50 mM Tris, pH7.5, 150 mM Nacl, 1% NP-40, and 1 mM ethylennediaminetetraacetic acid) containing phosphatase inhibitor cocktail (Roche Diagnostics, Indianapolis, IN) and protease inhibitor cocktail (Roche Diagnostics). Cells were incubated on ice for 30 minutes and collected by centrifugation at 10,000 g for 10 minutes. Cell lysates were separated by SDS-PAGE (9% polyacrylamide) and transferred onto nitrocellulose membranes. After blocking with 5% skim milk for 1 hour, the membranes were incubated with anti-MCP11 (R&D system, Minneapolis, MN) and anti-β-actin (Cell Signaling, Billerica, MA) overnight at 4 °C, followed by incubation with HRP-conjugated secondary antibodies (Cell Signaling) for 1 hour at room temperature (RT). Signals were developed with West Save Up**™** (AbFrontier, Seoul, Korea) and visualized by LAS4000 mini (Fujifilm, Tokyo, Japan).

### T cell proliferation and cytokine assay

After 3 days of stimulation, cell pellets were collected for T cell proliferation analysis (MTT assay; Roche Applied Bioscience, Branchburg, NJ) according to the manufacturer’s instructions. Before adding MTT reagent, supernatants were removed and the levels of IL-4, IL-6, IFN-γ, and IL-17A were measured using a standard sandwich ELISA (BD biosciences).

### Generation of BM chimeric mice

Femurs and tibias were taken from CD11c-DTR mice or WT C57BL/6 mice. BM was flushed out with a syringe and passed through a 40 μm nylon mesh to generate a single cell suspension. After red blood cells were lysed with LCK buffer, the cells were re-suspended in pH 7.4 PBS. CD45.1 recipient B6 mice (6–8 weeks-old) were irradiated with X-ray irradiator. The mice received two split doses at 450 cGy each before 10 × 10^6^ BM cells were administered intravenously. Chimeric mice were rested for at least 6 weeks before experiments^37^.

### DC depletion with diphtheria toxin

For depletion of CD11c^+^ cells *in vivo*, CD11c-DTR chimeric mice received daily intraperitoneal injections of DT (Sigma, St Louis, MO) at 8 ng/g in PBS for 7 to 10 days before immunization^37^. The efficiency of CD11c^+^ DC depletion was analyzed in peripheral blood.

### Immunization with CTO

For immunization, *Mcpt8*-cre-YFP mice and *Mcpt8*-cre × ROSA DTα mice were challenged intranasally with 40 μg of OVA_323-339_ peptide with 2 μg of CT (List Biological laboratories) in PBS^32^. One week after immunization, mice were anesthetized and BAL was performed and spleen, mediastinal LNs, and lung tissues were removed for cytokine analysis.

### Histological, cytological, and morphometric analyses

Target LNs were sampled and LN volumes were measured as previously described^48^. LNs were fixed in 4% paraformaldehyde for 4 hours, incubated in 20% sucrose solution for 2 hours at 4 °C, and embedded in tissue freezing medium. Serial sections of 10 μm thickness were cut parallel to the long axis of the LNs. After blocking with 5% donkey serum (Jackson ImmunoResearch, West Grove, PA) in PBST for 1 hour at RT, the central sections of each LN were incubated with one or more of the following primary antibodies overnight at 4 °C or 2 to 4 hours at RT; anti-mouse VEGFR3 (goat polyclonal, R&D Systems) and/or anti-mouse CD3ε (hamster monoclonal 145-2C11, BD biosciences). After several washes with PBST, sections were incubated for 2 hours at RT with one or more secondary antibodies: Cy3-conjugated anti-goat Ab or Cy5-conjugated anti-hamster Ab (all from Jackson ImmunoResearch). The samples were washed with PBS and mounted with fluorescence mounting medium (Dako, Carpinteria, CA). Fluorescent signals were visualized and digital images obtained using a Zeiss ApoTome inverted microscope or LSM 780 confocal microscope equipped with argon and helium-neon lasers (Carl Zeiss, Oberkochen, Germany).

### Intracellular cytokine staining

Single cell suspensions from spleen, mediastinal LNs and lung tissues were re-stimulated with PMA (50 ng/ml) and ionomycin (500 ng/ml) for 5 hours at 37 °C in the presence of Brefeldin A (10 μg/ml). After staining with EMA, Fc block (5 μg/ml) and antibodies against cell surface proteins, FITC-labeled anti-CD3 (clone 500A2) and APC-labeled anti-CD4 (clone GK1.5), cells were treated with Fix-Perm and Perm-Wash solutions (BD biosciences) and stained with intracellular antibodies against PerCP-Cy5.5-labeled anti-IFN-γ (clone XMG1.2), PE-labeled anti-IL-4 (clone 11B11), and Alexa-Fluor700-labeled anti-IL-17A (clone TC-11-18H10.1). Flow cytometry was performed with an LSRFortessa (BD biosciences) and data were analyzed with FlowJo software (Treestar, Ashland, OR).

### Experimental autoimmune encephalomyelitis

*Mcpt8*-cre-YFP mice and *Mcpt8*-cre × ROSA DTα mice were immunized in four sites on the back with 200 μg MOG_35-55_ peptides in 1 mg/ml CFA containing *M. tuberculosis* (Sigma-Aldrich, St Louis, MO). All mice also received 200 ng pertussis toxin (List Biological Laboratories) intraperitoneally on day 0 and 2. Mice were measured clinical scores for EAE according to the following scale: 0, normal; 1, paralyzed tail; 2, impaired reflex and hind limb weakness; 2.5, one hind limb paralyzed; 3, both hind limbs paralyzed; 3.5, hind limbs paralyzed and weakness in fore limbs; 4, fore limbs paralyzed; 5, moribund.

### Adoptive transfer of BMBs

After BM culture, WT and IL-6 deficient CD49b(DX5)^+^/c-kit^−^ basophils were isolated using a FACSAria II. Isolated basophils were injected intravenously into IL-6 deficient mice. The next day, mice were challenged intranasally with 40 μg of OVA peptides with 2 μg of CT. Inflamed lungs and draining LNs were collected after 7 days.

### Statistical analyses

Data are presented as mean ± SEM. P values were calculated using a two side Student’s t-test and are indicated with *(P < 0.05), **(P < 0.01), ^#^(P < 0.001). All analyses were performed using GraphPad Prism Software V. 6.0 for Windows (GraphPad Software, La Jolla, CA).

## Additional Information

**How to cite this article**: Yuk, C. M. *et al*. Basophil-derived IL-6 regulates T_H_17 cell differentiation and CD4 T cell immunity. *Sci. Rep.* **7**, 41744; doi: 10.1038/srep41744 (2017).

**Publisher's note:** Springer Nature remains neutral with regard to jurisdictional claims in published maps and institutional affiliations.

## Supplementary Material

Supplementary Information
