# Supplementary material for: Basophil-derived IL-6 regulates TH17 cell differentiation and CD4 T cell immunity
Source: Sci Rep. 2017 Jan 30;7:41744. doi: 10.1038/srep41744 (PMC5278410; doi:10.1038/srep41744)
Supplement: Supplementary Information [file srep41744-s1.pdf]

Supplemental Information

Basophil-derived IL-6 regulates T_H17 cell differentiation and CD4 T cell immunity

Chae Min Yuk, Hyeung Ju Park, Bo-In Kwon, Sang Joon Lah, Jun Chang, Ji-Young Kim, Kyung-Mi Lee, Su-Hyung Park, Seokchan Hong, and Seung-Hyo Lee

*Correspondence and requests for reprints should be address to:

Seung-Hyo Lee, PhD

Cellular Immunology Laboratory, GSMSE, KAIST, Daejeon, 34141 Korea

Phone: 82-42-350-4235, Fax: 82-42-350-4240, E-mail: sl131345@kaist.ac.kr

or

Seokchan Hong, MD/PhD

Division of Rheumatology, University of Ulsan College of Medicine, Asan Medical Center, Seoul, 05505 Korea

Phone: 82-2-3010-1410, Fax: 82-2-3010-6969, E-mail: medivineluke@gmail.com

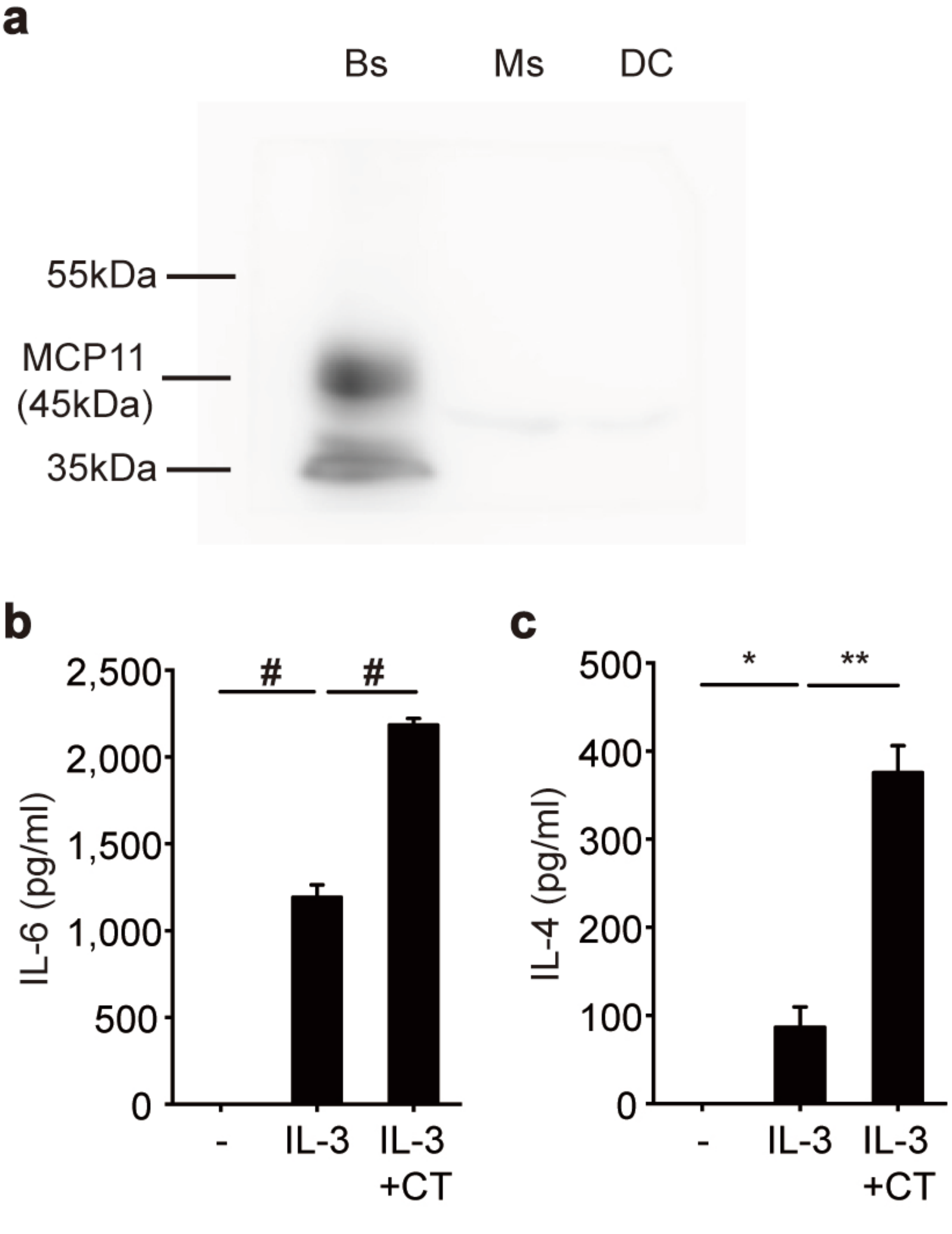

Supplemental Figure S1. IL-6 and IL-4 production by SPBs stimulated with IL-3 or IL-3 plus CT.

(a) Full-length blots of BMBs, BMMCs and BMDCs are presented for MCP-11 protein expression in Figure 1d. FACS-sorted YFP+ SPBs were stimulated with IL-3 or IL-3 plus CT for 24 hours. Secretion of IL-6 **(b)** and IL-4 **(c)** was quantified. Data are representative of three independent experiments (n=3).

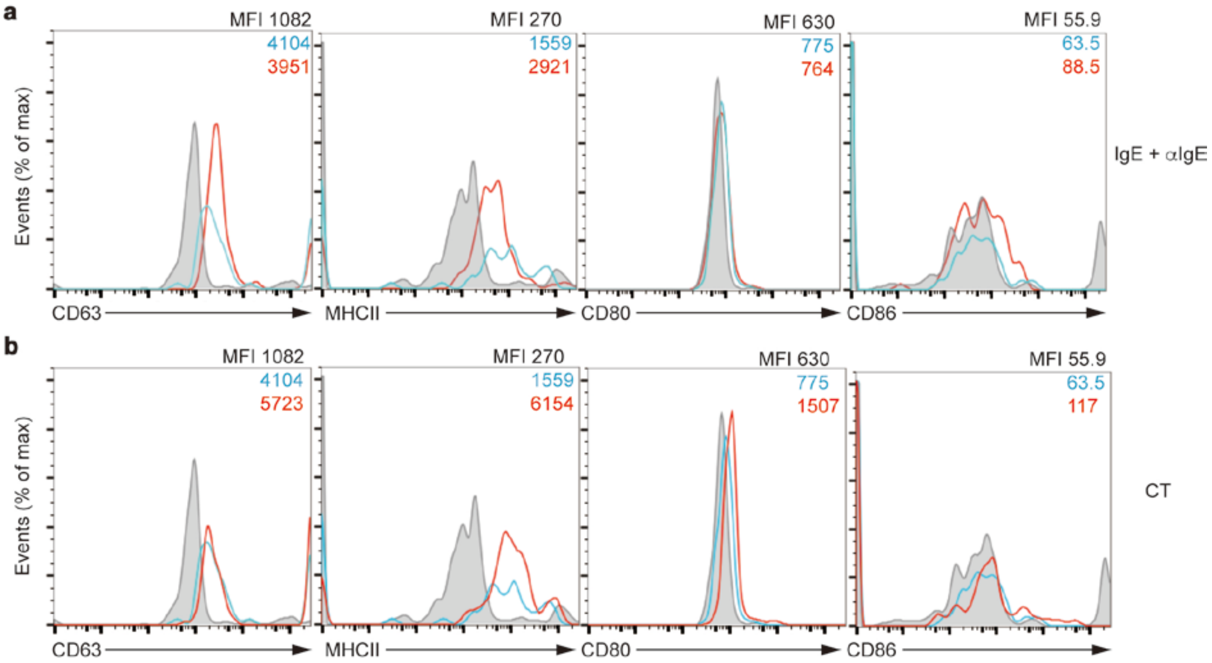

Supplemental Figure S2. Expression of cell surface markers on SPBs

Flow cytometric analysis of CD63, MHC class II, CD80, and CD86 expression on YFP^{+} SPBs were examined after IgE crosslinking **(a)** or incubation with CT **(b)** (red line) or without stimulation (blue line) for 2 hours. Grey bars represent staining with isotype control antibody. Data are representative of three independent experiments.

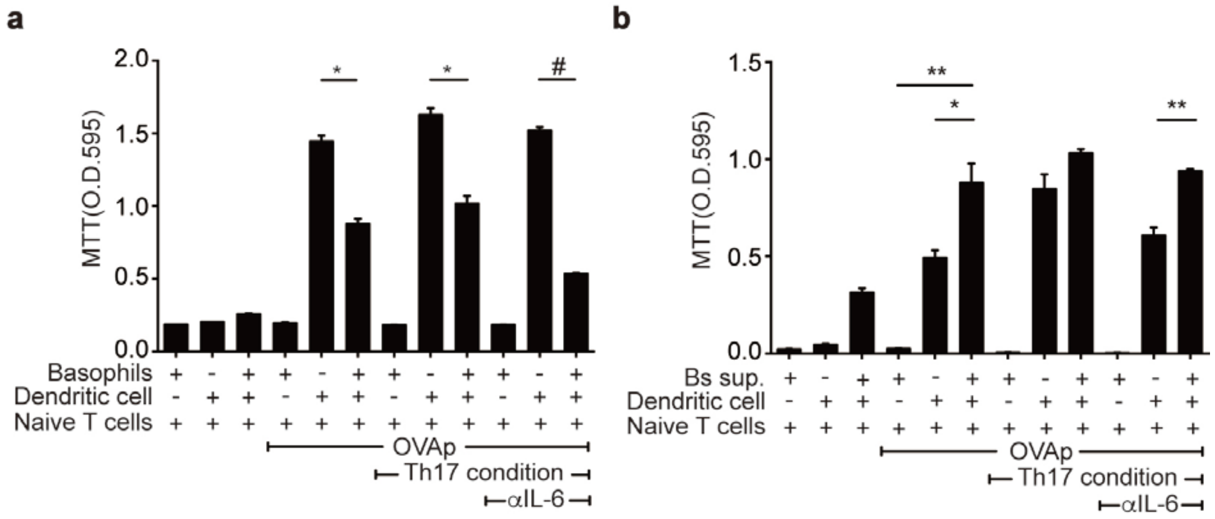

Supplemental Figure S3. Cooperation of basophils and DCs to drive T_H17 cell proliferation

Proliferation of OT-II CD4 T cells incubated with DCs, basophils, both DCs and basophils **(a)** or supernatants of activated basophils **(b)** was assessed with MTT assay as in Figure 4. Data are representative of three independent experiments (n=3) and are presented as mean ± SEM.

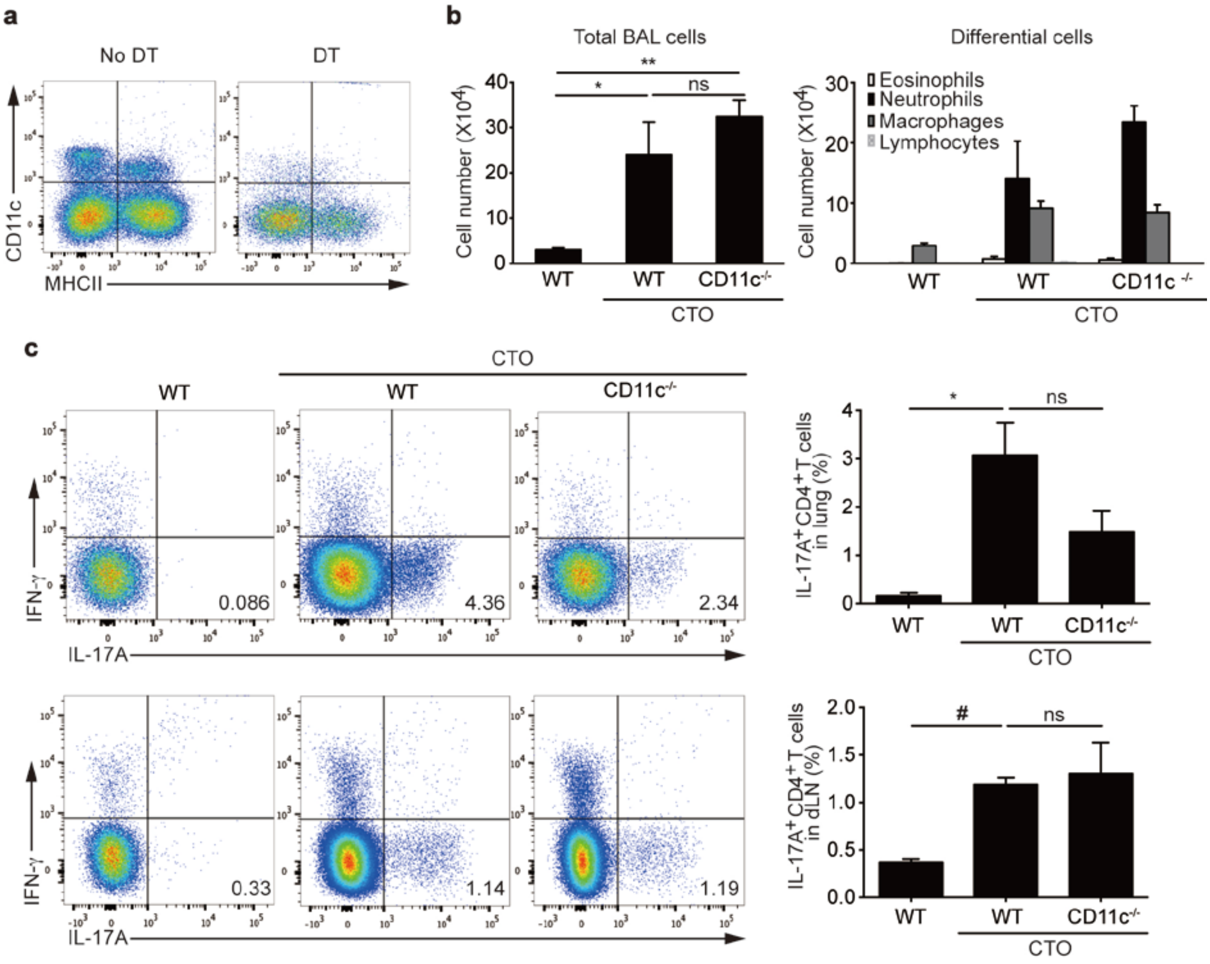

Supplemental Figure S4. Normal induction of TH17 responses in the absence of CD11c DCs

(a) CD11c+ DCs were depleted in CD11c-DTR mice by DT treatment. DC depletion was confirmed by FACS analysis. **(b)** After administration of CTO, the numbers of total cells and inflammatory cells in BAL fluid were calculated in each group. **(c)** Intracellular cytokine staining of CD4 T cells from lungs and mediastinal LNs of CTO-immunized WT and DC depleted mice were assessed for the production of IL-17A and IFN-γ. Data are representative of three independent experiments (n=3) and are presented as mean ± SEM.
